# [^18^F]Fluoro-2-Deoxy-D-Glucose Incorporation by MCF-7 Breast Tumour Cells *In Vitro* Is Modulated by Treatment with Tamoxifen, Doxorubicin, and Docetaxel: Relationship to Chemotherapy-Induced Changes in ATP Content, Hexokinase Activity, and Glucose Transport

**DOI:** 10.1155/2011/874585

**Published:** 2010-10-26

**Authors:** R. I. Sharma, A. E. Welch, L. Schweiger, S. Craib, T. A. D. Smith

**Affiliations:** ^1^School of Medical Sciences (Biomedical Physics), Aberdeen Biomedical Imaging Centre (ABIC), University of Aberdeen, Foresterhill, Aberdeen AB25 2ZD, UK; ^2^John Mallard PET Centre, Aberdeen Biomedical Imaging Centre (ABIC), University of Aberdeen, Foresterhill, Aberdeen AB25 2ZD, UK

## Abstract

Breast tumours responding to chemotherapy exhibit decreased [^18^F]fluoro-2-deoxy-D-glucose ([^18^F]FDG) incorporation. Underlying mechanisms of these changes is poorly understood. Here, in MCF-7 cells, responding to chemotherapy drugs commonly utilised in the treatment of breast cancer, [^18^F]FDG incorporation and several pivotal factors associated with [^18^F]FDG incorporation investigated. *Methods*. IC50 and subclinical doxorubicin, docetaxel, and tamoxifen doses determined using MTT assay. [^18^F]FDG incorporation by cells treated with IC50 drug doses for 48 hours and 72 hours were determined and FDG dephosphorylation estimated by measuring loss of 18F from [^18^F]FDG-preincubated cells (pulse-chase). Glucose transport determined by measuring initial uptake rate of non-metabolised glucose analogue omethylglucose; hexokinase activity and ATP content measured in cell homogenates; Cell cycle distribution determined using flow cytometry of propidium iodide stained nuclei. *Results*. [^18^F]FDG incorporation and ATP content decreased in cells after 72 hours treatment with IC50 doses of tamoxifen, doxorubicin, and docetaxel compared with untreated controls. Decreased glucose transport and/or hexokinase activity accompanied decreased [^18^F]FDG incorporation by MCF-7 cells treated with tamoxifen or doxorubicin but not docetaxel. *Conclusions*. Tumour cell [^18^F]FDG incorporation along with ATP content decreased by treatment with tamoxifen, doxorubicin and docetaxel paralleling clinical observations for solid tumours. Effect of each treatment on glucose transport and hexokinase activity was chemotherapy-drug dependent.

## 1. Introduction

Breast cancer is the most common form of neoplasia in women accounting for almost a third of all new cases of women's cancer [1]. Locally advanced breast cancer is currently treated using neoadjuvant therapy consisting of anthracyclines such as doxorubicin or taxanes [2, 3] including docetaxel. Tamoxifen, an antioestrogen, is commonly used for the adjuvant treatment of postmenopausal women presenting with early breast cancer. 

Both short-term and long-term toxicities are associated with the use of [2] adjuvant chemotherapy. Tamoxifen has side effects including hot flushes and vaginal discharges and other more serious side effects including thromboembolic disorders [4], and there are a number of novel aromatase inhibitors with more favourable tolerability [5]. Aromatase inhibitors act by inhibiting the synthesis of oestrogen from adrenal androgens in peripheral tissue thus reducing its level in the circulation of postmenopausal women in whom this is the major source of oestrogen. 

In view of the cytotoxic affects of adjuvant chemotherapy and tamoxifen treatment, early response indicators have been sought including the use of [^18^F]Fluoro-2-deoxy-D-glucose positron emission tomography ([^18^F]FDG-PET) [6–8]. Serial PET scanning of patients with breast cancer has shown that tumour response to chemotherapy is generally accompanied by decreased incorporation of [^18^F]FDG [6–8]. However, tumours of patients treated with tamoxifen have been shown to exhibit increased [^18^F]FDG uptake [9]. 

FDG enters cells by passive and facilitated diffusion. The latter is carried out by a family of glucose transporter molecules called gluts which exhibit altered expression in tumour cells compared with normal tissue [10]. On entering the cell, [^18^F]FDG is rapidly phosphorylated by hexokinase to [^18^F]FDG-phosphate. Due to the near absence from tumour cells of glucose-6-phosphatase [11, 12] and [^18^F]FDG-phosphate being a poor substrate for the next enzyme in the glycolytic pathway, FDG becomes trapped in the cell as [^18^F]FDG-phosphate. The extent to which modulation of these steps by chemotherapy contributes to therapy-induced changes in [^18^F]FDG incorporation at the tumour cell level is poorly understood but would be useful in the interpretation of clinical studies. One previous study of [^18^F]FDG uptake by MCF7 cells examined the effect of a 24-hour exposure of 1 *μ*mol/L doxorubicin on the gene and protein expression of hexokinase II and glut1 [13]. Their results showed that whilst glut1 expression was increased during exposure, there was no change in glut1 expression at the protein level. HKII expression was found to be diminished at both the mRNA and protein levels. Both glucose transporters [10, 14] and hexokinases [15] are highly regulated, so protein expression may not necessarily reflect functional activity

In this study, we have determined whether or not treatment with IC_50_ doses of tamoxifen, doxorubicin, or docetaxel modulate [^18^F]FDG incorporation at the tumour cell level and how these changes relate to HK activity and glucose transport, at the functional level, dephosphorylation/efflux. Decreased ATP levels are associated with drug response [16–18] and are likely to influence phosphorylation of [^18^F]FDG so we also determined ATP content during tumour cell response. Some regions of tumours are poorly perfused so drug levels that tumour cells are exposed to in those regions may be quite low. In view of this, we have also measured [^18^F]FDG uptake by cells treated with subclinical doses of chemotherapy agents to determine if changes in [^18^F]FDG uptake occur in the absence of appreciable tumour growth inhibition.

## 2. Materials and Methods

All chemicals were obtained from Sigma-Aldrich (Poole UK) unless otherwise stated.

### 2.1. Cells and Treatments

MCF-7 cells, an oestrogen-receptor positive breast cancer line, were cultured in DMEM (Invitrogen, Paisley, United Kingdom), and supplemented with 10% foetal bovine serum (FBS), 50 units/ml penicillin, and 50 *μ*g/ml streptomycin.

### 2.2. Cell Viability

Cells were grown to confluency in 75 cm^2^ flasks and after trypsinising and addition of medium the cell number was corrected to 15,000 per ml. One hundred microlitres of cell suspension was seeded into 96 well plates and the plates incubated at 37°C overnight. Media (100 *μ*l), supplemented with varying concentrations of the chemotherapeutic agents to produce final concentrations within the following ranges: tamoxifen (0.1–20 *μ*M); doxorubicin (0.001–0.1 *μ*M); docetaxel (0.00001–0.001 *μ*M), was then added to each well (200 *μ*l/well in total) and the plate returned to the incubator. A background of 200 *μ*l of media and a control of 200 *μ*l of media and cells were also set up. After either 48 or 72 hours, 50 *μ*l of MTT was added to each of the wells and incubated for 3-4 hours. The media was then aspirated using a syringe, and 200 *μ*l of DMSO (dimethylsulfoxide) was then added to each well. Each plate was then placed in a scanning multiwell spectrophotometer (Dynatech MR5000, Dynatech Labarotaries Inc., Chantilly, VA, USA) and agitated for 30 seconds prior to measuring spectrophotometric absorbance at 570 nm (test filter 570 nm, reference filter 690 nm). The plates were analysed using Biolinx 2.0 software (Biolinx 2.0, Dynatech Labarotaries Inc.).

### 2.3. [^18^ *F*]FDG Incorporation

Cells were seeded in 25 cm^2^ tissue culture flasks and 3 days later treated with IC_50_ and subclinical doses of tamoxifen (10 *μ*M and 0.05 *μ*M, resp.), doxorubicin (0.1 *μ*M and 0.0025 *μ*M, resp.), and docetaxel (0.5 nM and 0.001 nM, resp.). At the time of [^18^F]FDG uptake determination, medium was replaced with 1 ml of fresh medium (glucose concentration 1 mg/ml (5 mM) which is the level of glucose in the plasma of normal subjects) containing 37KBq [^18^F]FDG and incubated for 20 minutes at 37°C. Cells were then washed 5 times with 5 ml of ice cold PBS and detached by addition of trypsin. After addition of media to neutralise the trypsin, 0.2 ml was prepared for cell cycle analysis, and 0.8 ml of cell suspension was transferred into Eppendorf tubes for determination of [^18^F]FDG uptake in a well counter. The cells were then centrifuged, washed with 1 ml of PBS, the pellet was dissolved in 0.1 ml NaOH (1 M) and after neutralising with 0.1 ml HCl, protein content was determined.

### 2.4. *[^18^F] *FDG Release

[^18^F]FDG cellular release was determined by the modified method of Caracó et al. [12]. Cells treated and incubated with [^18^F]FDG as described above were washed 5x with 5 ml of ice-cold PBS. Cells were then incubated in 1 ml of fresh media for 5 minutes to allow efflux of non-phosphorylated [^18^F]FDG. Cells were then incubated in 1 ml of fresh media for 20 minutes. After removal of the medium and detachment of the cells, activity in the media and cells was determined. Activity effluxed was expressed relative to incorporated activity.

### 2.5. Glucose Transport

Glucose transport rate was determined by incubating cells with [^3^H] o-methyl-glucose ([^3^H] OMG) and the amount taken up during the initial linear phase of uptake considered to be a measure of glucose transport, which was determined by [19]. 

Flasks of cells were seeded as for FDG uptake and treated with IC_50_ doses of tamoxifen (10 *μ*M), doxorubicin (0.1 *μ*M), and docetaxel (0.5 nM) for 72 hours. The media was then discarded and replaced with fresh media (glucose concentration 1 mg/ml) containing 37KBq of [^3^H] OMG and 0.1 mM “cold” OMG for 10 seconds ([^3^H] OMG uptake by MCF-7 cells is linear for at least 30 seconds [11]) at 37°C. The incubation was ended by rapid addition of 5 ml of ice-cold PBS containing the glucose transport inhibitor phloretin (0.1 mM) followed by 3 further rapid washes. Cells were then trypsinised, half of them were added to 5 ml of Optima Gold scintillation fluid (Perkin Elmer, United Kingdom), and [^3^H] OMG uptake was determined in a scintillation counter. The remaining cells were prepared for protein assay.

To determine nonfacilitated transport of [^3^H] OMG further, flasks of cells were coincubated with [^3^H] OMG and 250 mM OMG to block facilitated uptake.

### 2.6. Hexokinase Activity

The preparation of cellular homogenates and the hexokinase assay was based on that of Miccoli et al. [20]. Cells were seeded in 75 cm^2^ flasks and after 3 days treated with IC_50_ doses of tamoxifen (10 *μ*M), doxorubicin (0.1 *μ*M), and docetaxel (0.5 nM) for 72 hours then harvested by trypsinisation. After addition of medium, they were collected into Eppendorf tubes, washed with PBS, and centrifuged at 400 g for 1 minute, and the pellet was resuspended in 0.2 ml homogenisation buffer (10 mM Tris/HCl, pH7.7 0.25 mM sucrose, 0.5 mM dithiothreitol, 1 mM aminohexanoic acid, and 1 mM PMSF). They were then transferred to a 1 ml glass dounce homogeniser and homogenised by 10 strokes at 4°C. The homogenised cells were transferred to an Eppendorf tube and centrifuged for 10 minutes at 1000 g to remove cell debris. The supernatant was transferred to a new Eppendorf tube, and the pellet was washed with 0.2 ml of homogenisation buffer, and the supernatant pooled. Protein content of the homogenate was determined on a 20 *μ*l sample.

Hexokinase enzyme protein level was determined by the addition of 100 *μ*l of homogenate to 0.9 ml of assay medium consisting of 100 mM Tris/HCl (pH 8.0), 10 mM glucose, 0.4 mM NADP^+^, 10 mM MgCl_2_, 5 mM ATP, and 0.15 unit of glucose-6-phosphate dehydrogenase in a cuvette at 37°C. The reaction was followed by monitoring the change in absorbance at 340 nm due to the formation of NADPH.

### 2.7. ATP Content

ATP content of treated and untreated cells was determined using a commercial kit (Sigma-Aldrich, Poole, UK) following the manufacturer's instructions.

### 2.8. Flow Cytometry

Flasks of cells were seeded as for FDG uptake and treated with IC_50_ doses of tamoxifen (10 *μ*M), doxorubicin (0.1 *μ*M), and docetaxel (0.5 nM) for 72 hours. Cells were harvested by tryspinization and collected into Eppendorf tubes. They were then centrifuged at 1000 g for 5 minutes and after removing the supernatant the pellet was washed with PBS and the pellet fixed by the slow addition of 1 ml of ice-cold 70% ethanol and left at −20°C for at least 24 hours. The fixed cells were then washed with PBS containing 1% serum then resuspended in staining buffer at 4°C for at least 20 minutes prior to analysis. The cell number was then determined using a haemocytometer and the cell concentration was adjusted to 1 × 10^6^ cells in a volume of 0.5 ml of propidium iodide (PI)/RNase staining buffer and the suspension was incubated for 15 minutes at room temperature. The stained cells were kept at 4°C and protected from light. Flow cytometry was performed using 488 nm lazer light on a FACSCalibur flow cytometer (Becton Dickinson) and flowjo software (Treestar) with doublet discrimination.

### 2.9. Statistics and Replicates

Significant differences between means were established using the Student *t*-test. The *t* value and corresponding level of significance is shown in brackets, for example, (*t* = 3, *P* < .05); if the difference between the means is not significantly different, this is written as (*t* = 3, ns).

## 3. Results

### 3.1. Cell Viability

**Figure 1:**
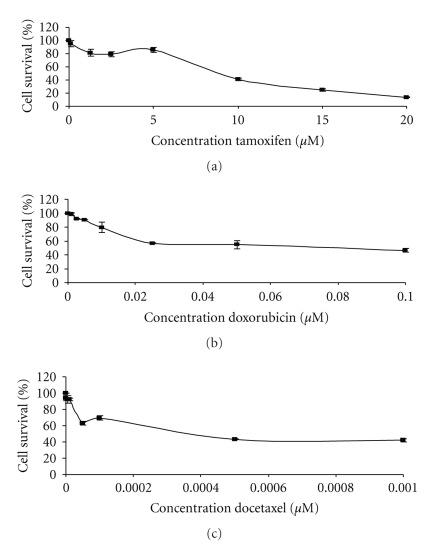
Cell viability determined by the MTT assay. Effect of tamoxifen (a), doxorubicin (b), or docetaxel (c) for 48 hours (black diamonds) and 72 hours (white squares) on MCF-7 tumour cell growth determined using the MTT assay and expressed relative to untreated cells. Each determination was carried out 3 times.

Figure 1 shows the decrease in cell number, determined using the MTT assay after treatment of MCF7 cells for 48 and 72-hour. Drug concentrations that produced about a 50% decrease in cell number after 72 hours treatment (IC_50_) were tamoxifen 10 *μ*M; Doxorubicin 100 nM; docetaxel 0.5 nM.

### 3.2. Flow Cytometry

**Figure 2:**
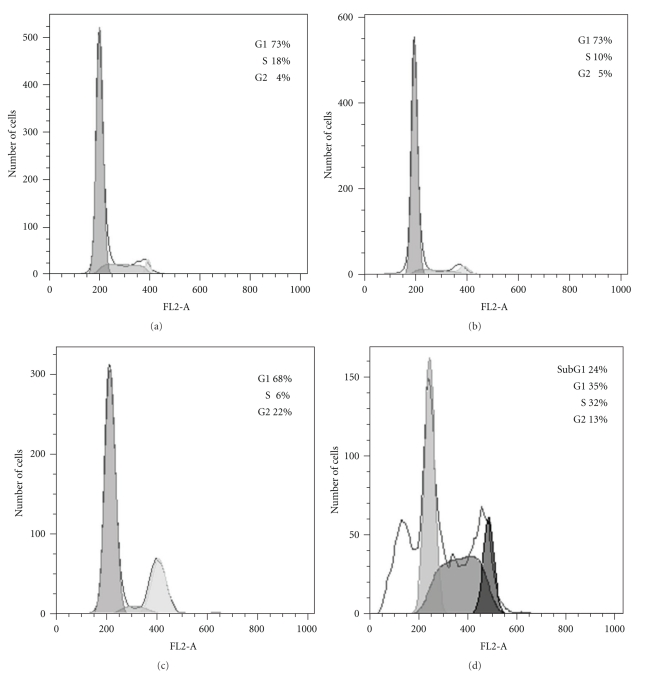
Cell cycle distributions determined on control and treated MCF-7 cells. Control (a) and MCF-7 cells treated with Tamoxifen (b), Doxorubicin (c), and docetaxel (d).

Cell cycle distributions for control and treated MCF7 cells are shown in Figure 2. Treatment of MCF-7 cells with tamoxifen was found to result in a decrease in S-phase cells compared with controls (*t* = 2.94, *P* < .05) whereas treatment of cells with doxorubicin resulted in a buildup of cells in G2. A subG1, indicative of an apoptotic population, was not evident in adherent cells after either tamoxifen or doxorubicin. However, a subG1 peak was associated with about 25% of cells treated with docetaxel. DNA analysis was also carried out on MCF-7 cells after 48 hours treatment (results not shown) and showed very similar cell cycle distributions to that of MCF-7 cells treated for 72 hours.

### 3.3. [^18^F]FDG Incorporation

**Figure 3:**
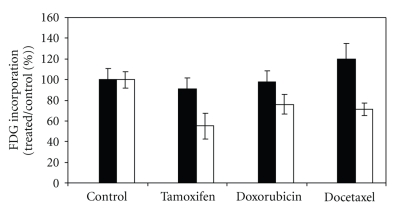
FDG incorporation by control and MCF-7 cells treated for 48 hours (black, *n* = 5 replicates) or 72 hours (white *n* = 10 or more replicates) with tamoxifen, doxorubicin, or docetaxel as a percentage of incorporation by untreated cells.

[^18^F]FDG uptake after treatment of MCF-7 cells with tamoxifen, doxorubicin, or docetaxel for 48 hours and 72 hours followed by incubation with [^18^F]FDG for 20min is shown in Figure 3. Compared with control cells treatment with tamoxifen (*t* = 1.22, ns) doxorubicin (*t* = 0.3, ns), or docetaxel (*t* = 2.13, ns) for 48 hours did not result in a significant change in FDG incorporation. Compared with control cells, [^18^F]FDG incorporation was significantly decreased by cells treated for 72 hours with tamoxifen (*t* = 10, *P* < .001) by 45%, doxorubicin (*t* = 6.4, *P* < .001) by 24%, and docetaxel (*t* = 9.6, *P* < .001) by 29%. 

The rate of efflux of ^18^F from cells incubated with [^18^F]FDG for 20 minutes was determined (results not shown). Compared with untreated cells, there was no significant difference in the rate of efflux of [^18^F]FDG from cells treated with tamoxifen or docetaxel. However, cells treated with doxorubicin exhibited a significantly (*t* = 9.35, *P* < .005) lower rate of ^18^F efflux compared with untreated cells.

Treating MCF-7 cells with subclinical amounts of tamoxifen or doxorubicin for 72 hours did not result in significant changes in [^18^F]FDG uptake. However, treatment with a subclinical dose of docetaxel caused a significant (*t* = 2.9, *P* < .01) decrease in [^18^F]FDG uptake although this change was less than 10% (results not shown).

### 3.4. Glucose Transport

**Figure 4:**
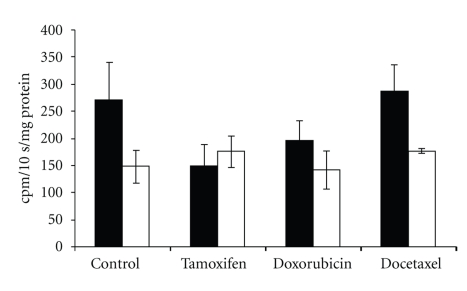
Glucose transport by control and treated MCF-7 cells determined by uptake of OMG during first 10 seconds of incubation of cells with 0.1 mM OMG (black) or 250 mM OMG (white).

Figure 4 shows the uptake of [^3^H] OMG by MCF-7 cells incubated with [^3^H] OMG for 10 seconds and either 0.1 mM OMG or a blocking concentration of 250 mM OMG. Transport of [^3^H] OMG was significantly reduced by the treatment of cells with doxorubicin (*t* = 3.01, *P* < .01) and tamoxifen (*t* = 4, *P* < .01) but not by docetaxel (*t* = 0.05, ns). The presence of 250 mM OMG reduced uptake of [^3^H] OMG, and there was no significant difference in uptake by control cells compared with uptake by tamoxifen-(*t* = 1.6, ns), doxorubicin (*t* = 0.35, ns) and docetaxel (*t* = 1.6, ns) suggesting that the rate of nonfacilitated uptake was the same by control and treated cells.

### 3.5. Hexokinase Activity

**Figure 5:**
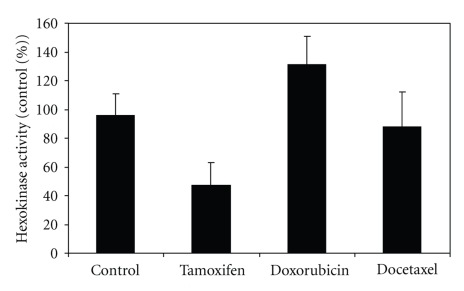
Hexokinase activity in MCF-7 cells treated for 72 hours with tamoxifen (*n* = 6), doxorubicin (*n* = 6), or docetaxel (*n* = 5) as a percentage of activity in untreated cells (*n* = 9).

Figure 5 shows hexokinase activity in MCF-7 control and in cells treated with tamoxifen, doxorubicin, and docetaxel. Tamoxifen treatment significantly (*t* = 3.06, *P* < .01) decreased HK activity whereas the treatment with doxorubicin actually increased HK activity (*t* = 4.37, *P* < .01). Treatment with docetaxel (*t* = 0.56, ns) did not significantly affect HK activity.

### 3.6. ATP Content

**Figure 6:**
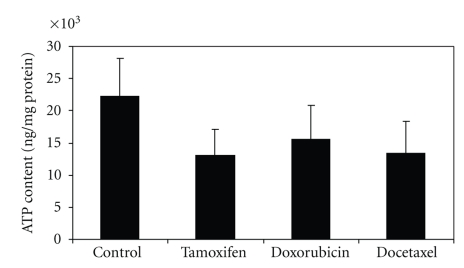
ATP content of MCF-7 cells treated for 72 hours with tamoxifen (*n* = 6), doxorubicin (*n* = 6), or docetaxel (*n* = 6) or untreated (*n* = 6).

Figure 6 shows ATP content in untreated and treated MCF-7 cells. Compared with control cells, ATP content was significantly decreased after treatment with tamoxifen (*t* = 3.7, *P* < .01), doxorubicin (*t* = 2.45, *P* < .02), and docetaxel (*t* = 3.33, *P* < .003).

## 4. Discussion

The findings of clinical [^18^F]FDG-PET studies have shown that the incorporation of [^18^F]FDG by breast tumours responding to adjuvant therapy is decreased compared with pretreatment uptake [6–8]. Here, we have determined if changes in [^18^F]FDG uptake can be seen at the tumour cell level and have shown that [^18^F]FDG incorporation by MCF-7 cells was decreased after 72 hours of treatment with tamoxifen, doxorubicin, and docetaxel. The extent to which FDG incorporation is decreased varied with treatment type with tamoxifen inducing almost a 45% decrease in uptake whereas doxorubicin decreased incorporation by less than 25%. Such differential effect between treatments suggests that the sensitivity of changes in FDG incorporation to detect tumour response may be chemotherapy agent dependent. 

Clinical studies of tamoxifen-treated breast tumours show an increase in metabolism at 1–2 weeks after starting treatment [9]. In this study, we found that treatment of both MCF-7 breast tumour cells for 72 hours with the IC_50_ dose of tamoxifen reduced [^18^F]FDG uptake. Clinical studies reporting a “metabolic flare” were performed shortly after patients started on a course of tamoxifen before blood concentrations would have achieved a growth-inhibitory level, and it has been shown that tamoxifen can be growth stimulating at low concentrations [9] perhaps accounting for the increase in [^18^F]FDG uptake observed after beginning tamoxifen treatment. Our findings suggest that when the tumour is regressing in response to therapy [^18^F]FDG uptake per tumour cell will be diminished. 

[^18^F]FDG incorporation has been shown to be a measure of cellular glucose utilization by brain tissue [21]. Studies [22] using ^13^C-NMR spectroscopy to probe ^13^C-glucose utilization have demonstrated that, compared to pretreatment, tamoxifen-treated MCF-7 cells exhibit a 50% decrease in glucose flux comparable to the decrease in [^18^F]FDG incorporation by MCF-7 cells in response to tamoxifen treatment reported in the present paper suggesting that [^18^F]FDG is measuring changes in glucose utilization in response to tamoxifen treatment. 

We have previously shown that MCF-7 cells express glucose transporters 1, 8, and 10 and that HK I is the principal HK in this cell line [11]. In the present study, HK activity was decreased in MCF-7 cells after treatment with tamoxifen whilst HK activity was enhanced in doxorubicin-treated MCF7 cells. However, the decrease in [^18^F]FDG incorporation by MCF-7 cells treated with tamoxifen or with doxorubicin paralleled decreased levels of glucose transport observed in this tumour line. Previously, it has been shown that hypoxia-induced increases in [^18^F]FDG uptake by MCF-7 cells has been shown to be associated with increased glucose transporter activity [23].

Decreased [^18^F]FDG incorporation by MCF-7 cells treated with docetaxel did not correspond with changes in glucose transport or HK activity. To determine whether or not treatment of MCF-7 cells increased the rate of dephosphorylation of [^18^F]FDG-phosphate, the rate of [^18^F]FDG efflux was determined in cells labelled with [^18^F]FDG. During the initial 5-minute incubation in fresh medium of cells preincubated with [^18^F]FDG about 10% of activity was lost from the cells (results not shown) consistent with our previous finding that 90% of [^18^F]FDG in MCF-7 cells is in the phosphorylated form [11] and in agreement with other workers [12]. During the subsequent 20-minute incubation, efflux was found to be appreciable despite the absence of expression of glucose-6-phosphatase from MCF-7 cells [11]. Dephosphorylation of [^18^F]FDG phosphate by cells not expressing glucose-6-phospatase has been described previously [12, 24] suggesting the presence of glucose-6-phosphatase-independent mechanisms for [^18^F]FDG-phosphate dephosphorylation. The rate of efflux of [^18^F]FDG from cells preincubated with [^18^F]FDG was modulated only in cells treated with doxorubicin. Thus, changes in dephosphorylation rate in docetaxel-treated cells could not account for the decrease in [^18^F]FDG incorporation compared with untreated cells.

Compared with untreated cells, ATP content was found to be decreased in MCF-7 cells treated for 72 hours with each drug. Decreased ATP content in toxin-treated cells including ones exhibiting apoptosis has also been reported previously [16–18]. In the absence of changes in other factors, the decrease in [^18^F]FDG incorporation by MCF-7 cells treated with docetaxel is likely to reflect decreased ATP content. 

Changes in cell cycle distribution of MCF-7 cells induced by tamoxifen or doxorubicin do not appear to be associated with the decrease in [^18^F]FDG uptake since although [^18^F]FDG accumulation was decreased by these agents, tamoxifen caused only minor changes in the cell cycle distribution with a small increase in G1 population whilst doxorubicin treatment caused an accumulation of cells in G2. This is in agreement with a previous paper [11], in which it was reported that treatment of MCF-7 cells with colchicine, which induces a buildup of cells in G2, was not associated with a change in [^18^F]FDG uptake. A further point is that changes in Cell cycle distribution after 48 hours of each treatment are similar to those seen after 72 hours. In contrast, the decreased FDG incorporation seen after 72 hours is not evident after 48 hours.

Docetaxel treatment induced apoptosis in adherent MCF-7 cells, however, the extent of decrease in [^18^F]FDG incorporation was similar to that of cells responding to treatment with doxorubicin and tamoxifen. Decreased [^18^F]FDG uptake by apoptotic tumour cell populations has recently been reported by Takei et al. [25], who showed that the decreased uptake of [^18^F]FDG in tumours derived from allogenic hepatoma cells grown in rats responding to treatment with gemcitabine and cyclophosphamide correlated with an enhanced apoptotic reaction. Takei et al. [25] did not observe any change in glut1 protein levels after treatment with either gemcitabine or cyclophosphamide. Similarly, we did not observe changes in glucose transport in MCF-7 cells treated with docetaxel. Increases in [^18^F]FDG incorporation could be expected to occur at early time points during therapy. Changes in mitochondrial membrane potential are associated with apoptosis, and we have previously shown that perturbation of the mitochondrial membrane can induce increases in [^18^F]FDG incorporation [26]. The difference in the uptake of [^18^F]FDG by cells treated for 48 hours (no change from controls) and 72 hours (decreased compared with controls) with chemotherapy agents illustrates the importance of the correct timing of posttreatment PET scans when using PET to measure response. 

As well as treating MCF-7 cells with IC_50_ drug concentrations, cells were also treated with subclinical doses of drugs, that is, a concentration that produced less than a 5% reduction in tumour cell number at 72 hours. We found no significant change in [^18^F]FDG uptake after treating cells with such doses of doxorubicin or tamoxifen, but treatment of MCF-7 cells with a subclinical dose of docetaxel resulted in a small but significant decrease in [^18^F]FDG uptake. This suggests that even very low levels of clinical response to some chemotherapy agents may be accompanied by changes in [^18^F]FDG uptake. 

In vivo, other factors would be expected to contribute to [^18^F]FDG incorporation including [^18^F]FDG delivery (blood flow), the presence of immune infiltrate which includes highly glucose utilizing macrophages [27], and areas of hypoxia and consequent HIF-1 activation [28], all of which will influence [^18^F]FDG. However, the purpose of this paper was to determine the effect of chemotherapy agents on [^18^F]FDG incorporation by tumour cells in the absence of other factors found in solid tumours. Our findings indicate that, at least after 72 hours of treatment, [^18^F]FDG incorporation is reduced during tumour response to chemotherapy and tamoxifen treatment paralleling the findings of decreased [^18^F]FDG incorporation by solid tumours responding to therapy reported in clinical studies. 

In conclusion, we have found that [^18^F]FDG incorporation by breast tumour cells in vitro is modulated by chemotherapy in a time-dependent manner. ATP content was decreased after 72 hours treatment with each agent corresponding with decreased [^18^F]FDG incorporation at this time point. Chemotherapy-induced changes in glucose transport and hexokinase activity were not consistently associated with decreased [^18^F]FDG incorporation.
